# Cancer Cell-Derived Exosomes Promote HCC Tumorigenesis Through Hedgehog Pathway

**DOI:** 10.3389/fonc.2021.756205

**Published:** 2021-10-07

**Authors:** Li Li, Jing Zhao, Quanbao Zhang, Yifeng Tao, Conghuan Shen, Ruidong Li, Zhengyu Ma, Jianhua Li, Zhengxin Wang

**Affiliations:** ^1^ Department of General Surgery, Huashan Hospital, Fudan University, Shanghai, China; ^2^ Institute of Organ Transplantation, Fudan University, Shanghai, China

**Keywords:** hepatocellular carcinoma, exosome, sonic hedgehog, tumorigenesis 3, cancer stem cell

## Abstract

**Purpose:**

Hepatocellular carcinoma (HCC) accounts for more than 80% of primary liver cancers and is one of the leading causes of cancer-related death in many countries. Cancer cell-derived exosomes are shown to mediate communications between cancer cells and the microenvironment, promoting tumorigenesis. Hedgehog signaling pathway plays important roles in cancer development of HCC.

**Methods:**

Exosomes were isolated from culture medium of HCC cell lines PLC/PRF/5 and MHCC-97H and were found to promote cancer cell growth measured with cell proliferation and colony formation assay. HCC cells cultured with cancer cell-derived exosome had increased cancer stem cell (CSC) population demonstrated by increased cell sphere formation CSC marker expressions. Hedgehog protein Shh was found to be highly expressed in these two HCC cell lines and preferably carried by exosomes. When Shh was knocked down with shRNA, the resulting exosomes had a reduced effect on promoting cancer cell growth or CSC population increase compared to normal cell-derived exosomes.

**Results:**

The ability of PLC/PRF/5 cells to form tumor in a xenograft model was increased by the addition of the exosomes from control cancer cells but not the exosomes from Shh knocked down cancer cells. Finally, the higher plasma Exo-Shh levels were associated with later tumor stages, higher histological grades, multiple tumors, and higher recurrence rates.

**Conclusion:**

This study demonstrated that HCC cells secreted Shh *via* exosome and promote tumorigenesis through the activated Hedgehog pathway.

## Introduction

Hepatocellular carcinoma (HCC), accounting for nearly 80% of the total number of liver cancers, is the fourth most common cause of death related to cancer (1). Surgical resection is still the primary choice for treatments. The nonoperative treatments include transcatheter arterial chemoembolization (TACE), radiofrequency ablation (RFA), and systematic administration of targeted therapy represented by sorafenib. However, despite the advancement in therapeutics, the median survival time for HCC is still only 6–20 months (2). Better understanding the biology of HCC and developing more therapeutic targets are still needed for improving the outcome.

The Hedgehog signaling pathway is a master regulator in animal development. First discovered in *Drosophila*, Hedgehog is a conserved morphogen secreted to regulate differentiation process in many metazoans. Subsequently, it is also found to play roles in maintenance of adult stem cells and in the progression of various diseases. The canonical Hedgehog pathway involves the binding of Hedgehog protein to a 12-transmembrane receptor PTCH1, releasing the G-protein-coupled receptor smoothened (SMO) from its inhibitory effect. Activated SMO in turn releases transcription factor GLI1 (glioma-associated oncogene family members) from its inhibitors SUFU and KIF7. The released GLI1 is translocated into nucleus and activates transcription of a number of genes, including GLI1, PTCH1, Cyclin D, Cmyc, and VEGF. In mammals, three homologs of Hedgehog proteins, sonic (Shh), Indian (Ihh), and desert (Dhh) have been identified, of which Shh is the most broadly expressed and asserts its function through paracrine or autocrine fashion.

Evidence for the role of Hedgehog signaling in cancer development has been revealed in many studies. Mutations in Hedgehog pathway components are often found in cancer. Ligand-independent activation of Hedgehog signaling due to inactivation mutations on negative regulator PTCH or hyperactivation of SMO or GLI1 was found in various cancers (3, 4).

Healthy adult hepatocytes do not express Hedgehog ligand. During injury or severe stress, liver epithelial cells would produce Hedgehog protein to activate the pathway. It was found that partial hepatectomy activates Hedgehog signaling in mice and that individuals with primary biliary cirrhosis have also upregulated signaling (5, 6). HBV or HCV infection would induce Hedgehog expression (7). The activated Hedgehog pathway is found associated with hepatocarcinogenesis (8). Aberrant activation of Hedgehog signaling pathway components including Shh, PTCH1, GLI, and SMO has been found in HCC (9–12). Shh is the most highly expressed Hedgehog ligand in HCC, expressed in about 60% of HCC patients and its presence is concentrated in and around tumor (13).

Extracellular vesicles (EVs) are lipid-bound particles derived from cells. They can be characterized into two types based on their size, content, biogenesis, and release pathways: microvesicles (MVs) and exosomes. MVs, usually at a diameter greater than 100 nm, are secreted by cells in the form of budding from the plasma membrane. Exosomes, usually at a diameter of 30–150 nm, are derived from intraluminal vesicles in polycystic bodies and are released by cells through the fusion of polycystic bodies and cell membranes. Both MVs and exosomes carry cargos including lipid, RNA, and proteins, and play important roles in regulating various biological processes.

Studies on the roles of EVs in cancer development have demonstrated its versatile functions in mediating communication between tumor and its microenvironment (14). EVs secreted by cancer cells have been subjects of studies as biomarkers and targets of therapy (15). In HCC, it has been found that exosome can induce various cellular biological changes in promoting cell proliferation, migration, angiogenesis, and immune suppression during tumorigenesis (16).

It is known that Hedgehog ligands undergo complicated post-translational modifications that results in lipid attachment and multimerization (17). It can be released in various forms, such as lipoproteins (17), or in association with extracellular vesicles (EVs) (18, 19). In the Drosophila model, the Hedgehog ligand carried on the surface of MV and exosome can activate the Hedgehog signaling pathway in promoting embryonic development (18).

In this presented study, we investigated the effect of exosomes secreted by HCC cell lines and demonstrated the evidence that HCC released Shh through exosome to promote cell proliferation and tumor formation.

## Materials and Methods

All human subjects involved in the study have signed informed consent for participation. The experimental protocol was reviewed and approved by the ethics committee of Fudan University. Peripheral blood samples were collected in tubes containing EDTA anticoagulation, stored at 4°C for no more than 8 h before centrifuging at 1,000 *g* for 10 min to obtain plasma. The plasma samples were stored at −80°C before further analysis. HCC tissue specimens were obtained during surgical resection. Samples were fixed in 4% paraformaldehyde and subjected to subsequent immunohistochemical analysis. Patients’ medical data were obtained through patients’ medical record with permission, and the post-surgical follow-ups were carried out in line with patients’ routine medical visit.

### Animal

Male NOD/SCID mice age of 6 weeks were obtained from SLAC Laboratory Animal Corporation (Shanghai, China). All animal experiments were performed according to animal protocol approved by animal care ethic committee of Fudan University. The xenograft tumor model was established by inoculating PLC/PRF/5 with or without Exo treatment subcutaneously into the groin of mice. Animals were continually monitored for 4 weeks before being euthanized. Tumors were collected and fixed in 4% paraformaldehyde. The tumors were weighted and measured, and the volumes were calculated based on the formula: V = 0.5*long diameter*short diameter^2^.

### Cell Lines

Hepatic carcinoma cell lines PLC/PRF/5 and MHCC-97H (abbreviated as PLC and 97H in figure label) and normal hepatic cell line L02 were obtained from Cancer Institute of Fudan University (Shanghai, China) and were maintained in DMEM cell culture medium (Gibco, New York, USA) supplemented with 10% FBS (Gemini, West Sacramento, USA) and 1% ampicillin and streptomycin in the incubator at 37°C with 5% CO_2_. The EV free FBS was obtained by centrifuging the purchased FBS at 120,000 *g* 4°C for 16 h, and filtering the supernatant through a 0.22-μm filter.

### Reagents

Primary antibodies used in the Western blot studies are as follows: anti-human Grp75, CD9, OCT4 (CST, Danvers, USA), anti-human ALDH, CD44, CD133 (GeneTex, Irvine, USA), anti-human sytenin1 (Abcam, Cambridge, UK), anti-human CD64 (Proteintech, Wuhan, China), and anti-human GAPDH [Beyotime Institute of Biotechnology (Nanjing, China)]. HRP conjugated secondary antibodies are from Beyotime Institute of Biotechnology (Nanjing, China). Growth factors bFGF and EGF were purchased from Gibco. MicroBCA protein quantification kit was purchased from Thermo Scientific (USA). Cell Counting Kit-8 (CCK-8) was purchased from Dojindo Laboratories (Kumamoto, Japan).

### Conditioned Medium

Cells grown to 60% confluency were washed three times with PBS before they are placed in the new medium supplemented with EV free FBS and continued to grow for another 24 h at 37°C with 5% CO_2_. The medium is collected and centrifuged at 300 g for 10 min, and the supernatant was further centrifuged at 2,000 *g* for 20 min. The resulting supernatant is the conditioned medium.

### Exosome Purification

EV and subsequent Exo and MV isolation from CM are based on Kowal et al. (20). Briefly, CM was centrifuged at 10,000 *g* for 40 min at 4°C. After supernatant was removed, the pellet, which was the MV fraction, was washed with PBS and resuspended in 50–100 µl of PBS. The supernatant from previous centrifugation was centrifuged again at 100,000 *g* for 90 min at 4°C. After the supernatant was discarded, the pellet, which was the Exo fraction, was washed with PBS and resuspended in 50–100 µl of PBS.

### Sphere Formation Assessment

A total of 250 PLC/PRF/5 or 500 MHCC-97H cells were seeded in 24-well plates in 1 ml of DMEM/F12 medium supplemented with 20 ng/ml epidermal growth factor (EGF), 20 ng/ml fibroblast growth factor (FGF), 2% B27, and 1% penicillin. The plate was shaken well before sealed with parafilm and cultured at 37°C, 5% CO2. Replenishment of the medium was made at 0.2 ml per well after 1 week of culture. Sphere count was carried out under the microscope after 2 weeks of culture. Each condition has three triplicate wells.

### Cell Proliferation Assay

PLC/PRF/5 and MHCC-97H cells were seeded in a 96-well plate at 1,000 or 2,000/well respectively, and incubated at 37°C and 5% CO_2_ for 24 h before various stimulus conditions were applied. Cells were continued to culture for another 48 h before the Cell Counting Kit-8 (CCK8) working solution, made by diluting CCK8 reagent in culture medium at 1:9 ratio, was added at 100 μl/well. Absorbance at 450 nm was measured by a spectrometer after 2 h. Five replicates were included for each culture condition.

### Clone Formation Assay

PLC/PRF/5 and MHCC-97H cells were seeded in 24-well plates at 125 or 250/well respectively, and incubated at 37°C, 5% CO_2_ for 24 h before various stimulus conditions were applied. Cells were continued to culture for another 10 days before the culture medium was removed. Cells were washed with PBS twice before fixed with 4% paraformaldehyde for 10 min and stained with crystal violet. After the plates were washed and air dried, the number of clones with more than 50 cells was counted.

### Protein Analysis

Total protein levels of extracted samples were determined with the Micro BCA Protein Assay Kit (Thermo Scientific). Protein samples were resolved with SDS-PAGE and transferred onto PVDF membrane for Western blot analysis. Primary antibodies were diluted according to the manufacturer’s instruction and incubated with membrane at 4°C, overnight with constant agitation. Secondary antibody incubations were carried out at room temperature for 1.5 h. ECL was used for visualization. Shh ELISA (Abcam) was performed as specified by the manufacturer.

### Lentivirus Infection

shRNA was introduced by shRNA carrying lentivirus made by GenePharma (Shanghai, China). The shRNA was cloned into GenePhama Supersilencing vector under CMV promoter/enhancer. The vector carries GFP gene for visualization and puromycin resistance gene for selection. Cells were grown in a six-well plate for 24 h to about 50% confluency. Cells were washed with PBS, and viral transfection solution was added together with 3 μl of Polybrene. Cells were incubated at 37°C, 5% CO_2_ for 24 h before changing into normal medium. Transfection efficiency was estimated by observing green fluorescence under a fluorescence microscope at 48 h post transfection. Puromycin were added at 48 h post transfection to maintain stable transfected cells. ShRNA sequences were as follows: Shh-shRNA1: GCTCGGTGAAAGCAGAGAACT, Shh-shRNA2: GCCAAGAAGGTCTTCTACGTG.

### RNA Analysis

Total RNA was extracted from cells using Trizol/chloroform method, and precipitated with isopropanol. Reverse transcription was performed according to the manufacturer’s instruction (Takara, Japan). Primers for qPCR (Takara, Japan) are listed in **Table 1**.

**Table 1:** Sequence of primers in quantitative PCR analysis.

Primer name	Primer sequence
Actin-F	5’-AATCGTGCGTGACATTAAGGAG-3’
Actin-R	5’-CAGGAAGGAAGGCTGGAAGAG-3’
Oct4-F	5’-ACCGAGTGAGAGGCAACC-3’
Oct4-R	5’-TGAGAAAGGAGACCCAGCAG-3’
CD133-F	5′-AGTCGGAAACTGGCAGATAGC-3′
CD133-R	5′-GGTAGTGTTGTACTGGGCCAAT-3′
CD44-F	5’-CTGCCGCTTTGCAGGTGTA-3’
CD44-R	5’-CATTGTGGGCAAGGTGCTATT-3’
ALDH1-F	5’-CCGTGGCGTACTATGGATGC-3’
ALDH1-R	5’-GCAGCAGACGATCTCTTTCGAT-3’
GLI1-F	5’-AGCGTGAGCCTGAATCTGTG-3’
GLI1-R	5’-CAGCATGTACTGGGCTTTGAA-3’
PTCH1-F	5’-CCAGAAAGTATATGCACTGGCA-3’
PTCH1-R	5’-GTGCTCGTACATTTGCTTGGG-3’
Cmyc-F	5’-GTCAAGAGGCGAACACACAAC-3’
Cmyc-R	5’-TTGGACGGACAGGATGTATGC-3’
CyclinD1-F	5’-TGGAGCCCGTGAAAAAGAGC-3’
CyclinD1-R	5’-TCTCCTTCATCTTAGAGGCCAC-3’

### Statistical Analysis

Statistical analysis was performed using SPSS 20 and GraphPad Prism 8 software. All experiments were performed with three biological replicates. Chi-square test was used in the comparison of the count data. Student’s *t*-test was used in the comparison of the measurement data between the two groups, while one-way ANOVA was used in the comparison of the measurement data among multiple groups. *p*-value < 0.05 indicates statistical difference, *<0.05, **<0.01, and ***<0.001.

## Results

### Exosome Purification

Exosome can be isolated based on its physical, chemical, and biological property, with methods mainly including ultracentrifugation, polymer precipitation, ultrafiltration, chromatography, and immunoaffinity capture. Among them, ultracentrifugation is more time-consuming. However, with relatively high purity in extraction, it is still a widely used method.

To understand whether EVs would play a role in HCC development, we extracted and separated EVs secreted by HCC cell lines PLC/PRF/5 and MHCC-97H, as well as the normal human liver cell line L02 using ultracentrifugation. Cells were cultured in DMEM supplemented with 10% exosome-free FBS before the isolation process. After a separation protocol containing ultracentrifugation at 10,000 *g* and 100,000 *g*, the EV was separated into MV and exosome (Exo) fractions.

**Figure 1:**
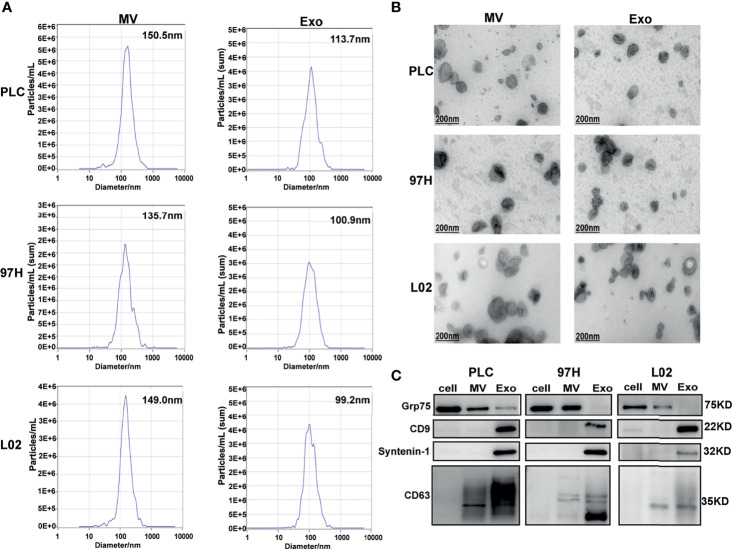
Examine the extracellular vesicles (EVs) isolated from HCC cell lines PLC/PRF/5 and MHCC-97H, as well as normal hepatic cell line L2. **(A)** Nanoparticle tracking analysis of the sizes of the EVs isolated from the three cell lines. **(B)** Electron microscope images of isolated EVs isolated from the three cell lines. **(C)** Western blot analysis of extracellular vesicle markers on the EVs isolated from the three cell lines.

The nanoparticle tracking analysis showed that the average particle sizes of the MV fractions from the three cell lines were between 135 and 150 nm in diameter, while the average sizes of the particles from the Exo fractions were between 99 and 114 nm in diameter (**Figure 1A**). Electron microscopy analysis showed that the extracted MV and Exo particles were in typical cup-shape morphology (**Figure 1B**). Analysis of particles and protein levels revealed that both PLC/PRF/5 and MHCC-97H cells secreted more particles as Exo than MVs, and these particles contained more protein in Exo than MVs (**Supplementary Figure S1**).

The purity of the fractions was analyzed by Western blot using protein markers specific for each fraction. It is shown that MV marker Grp75 was predominantly presented in the cell extract and MV fractions, while the Exo markers CD9, Syntenin-1, and CD63 were predominantly presented in the Exo fractions (**Figure 1C**). These results indicated that we successfully separated Exo and MV fractions through ultracentrifugation process, and this provided us the tools in studying the effect of Exo in subsequent experiments.

### Exosome Effect on Cancer Cell Growth

**Figure 2:**
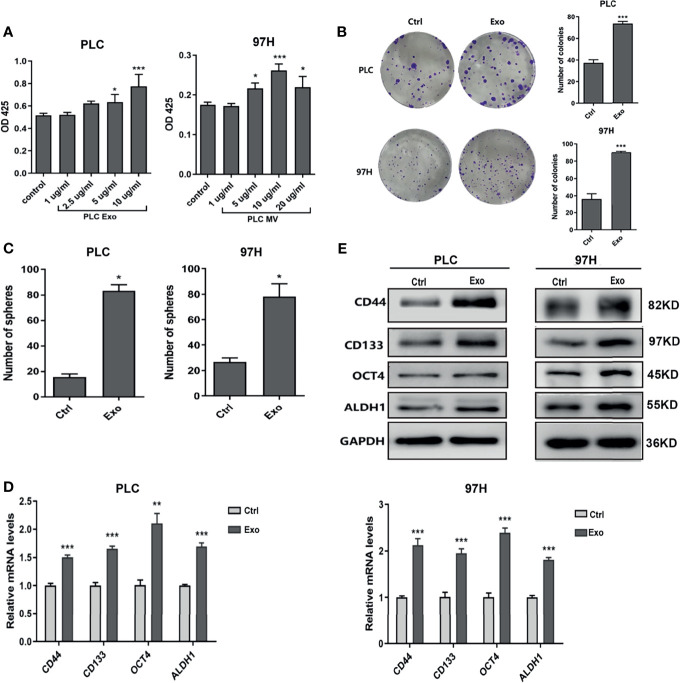
Effect of exosomes on HCC cell growth. **(A)** Proliferation of PLC/PRF/5 and MHCC-97H in the presence of various concentrations of exosomes isolated from culture medium of the same cell line as measured by CCK8 assay. **(B)** Colony formation assay and **(C)** count of sphere formed in PLC/PRF/5 and MHCC-97H with and without exosomes (10 μg/ml) isolated from the culture medium of the same cell line. **(D)** Quantitative PCR analysis of the mRNA levels, and **(E)** Western blot analysis of the protein levels of stem cell marker genes in PLC/PRF/5 and MHCC-97H cells cultured with and without exosomes (10 μg/ml) isolated from the culture medium of the same cell line. The data are presented as an average of three biological replicates. *p < 0.05, **p < 0.01, ***p < 0.001.

To determine whether exosome secreted by cancer cells has any effect on cell growth, we cultured PLC/PRF/5 and MHCC-97H cells in the presence of additional exosome isolated from their respective culture medium. As shown in **Figure 2A**, addition of Exo into the culture medium promoted PLC/PRF/5 and MHCC-97H growth in a dose-dependent manner. When the Exo concentration was above 5 µg/ml, the cell proliferations were significantly increased. We then studied the colony formation ability. When Exo is added at a concentration of 10 µg/ml in culture medium, the numbers of colonies formed were significantly increased in both PLC/PRF/5 and MHCC-97H (**Figure 2B**). We then investigated whether the addition of exosomes could lead to an increased subpopulation of cancer stem-like cells in culture. The morphology of cells cultured in medium supplemented with Exo were compared with those cultured in normal medium, and we found that the number of spheres in culture increased significantly in both cell lines when isolated Exo was added into culture medium (**Figure 2C**). The expression levels of several cancer stem cell (CSC) markers, CD55, CD133, OCT4, and ALDH1 were also increased at both transcription and protein level (**Figures 2D, E**) when cells were cultured with additional cancer cell-derived exosomes. These findings suggested that the exosome secreted by cancer cells was responsible for promoting cell growth and inducing CSC.

### Shh Signaling Is Responsible for the Effect Seen With Exosome

Several studies have shown that Hedgehog signaling pathway plays an important role in HCC growth and development. We decided to investigate whether the effect of exosome on cell growth was through the activation of the Hedgehog pathway.

**Figure 3:**
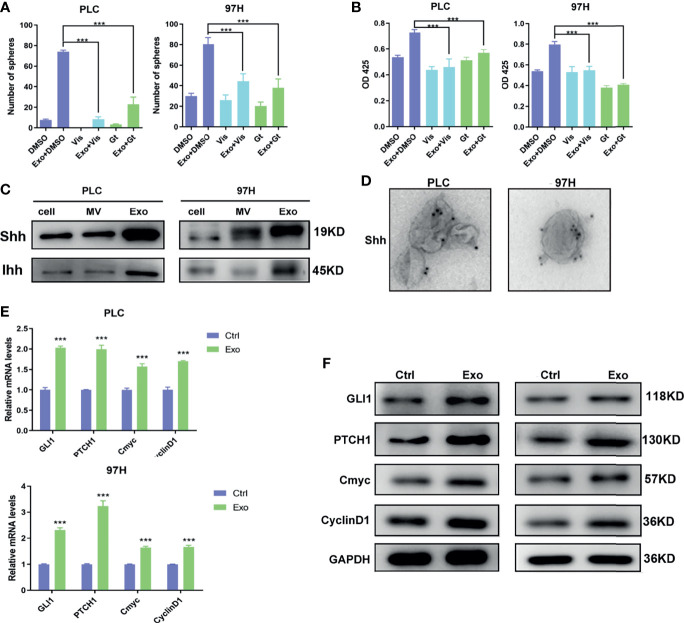
The role of Shh and hedgehog signaling pathway in promoting cell growth. The effect of hedgehog inhibitors on the stimulating effect of Exo (10 μg/ml) on **(A)** sphere formation and **(B)** cell proliferation. The inhibitors were added at the concentrations of 5 μM for GANT61 or 10 μM for Vismodegib. **(C)** The expressions of hedgehog ligands in PLC/PRF/5 and MHCC-97H as analyzed by Western blot. **(D)** Electron microscope image of immunogold staining of Shh on MV and Exo of PLC/PRF/5 and MHCC-97H. **(E)** Quantitative PCR analysis of the mRNA levels, and **(F)** Western blot analysis of the protein levels of hedgehog signaling pathway target genes in PLC/PRF/5 and MHCC-97H cells cultured with and without exosomes (10 μg/ml) isolated from the culture medium of the same cell line. The data are presented as an average of three biological replicates. ***p < 0.001.

Two inhibitors of the Hedgehog pathway, Gant-61, which inhibits GLI transcription factor, and Vismodegib, which inhibits SMO receptor, were added into the cell culture, and the effects of these inhibitors on Exo stimulation were studied. Both inhibitors, while themselves had minimal effects on cell proliferation or cell sphere formation, could significantly reverse the stimulating effects of the Exo on cell proliferation and sphere formation on both HCC cell lines (**Figures 3A, B**).

This promoted us to examine the expressions of Hedgehog protein in the two cancer cell lines. Two forms of Hedgehog proteins Shh and Ihh were expressed in these cell lines, with Shh being expressed at a higher level in both cells (**Figure 3C**). Hedgehog proteins were also found in EVs isolated from the cell culture, in which Exo contained much higher levels of both Shh and Ihh than MV. Both cancer cell lines expressed significantly higher Hedgehog protein in the secreted exosomes than the normal hepatocyte cell line L2 (S2), suggesting that the Hedgehog signaling was preferably activated in cancer development. Our results indicated that PLC/PRF/5 and MHCC-97H might use Shh as the main Hedgehog pathway ligand, and Exo would be the preferable way for the cells to secrete the protein.

We performed immunogold labeling with anti-Shh antibody on the exosomes isolated from PLC/PRF/5 and MHCC-97H cells, and as shown in **Figure 3D**, Shh was localized on the surface of Exo secreted by cancer cells. This indicated that Shh carried by exosome may directly interact with its receptor on cell membrane.

To study whether these Shh carrying exosomes can activate the Hedgehog pathway, we assess the expressions of the Hedgehog pathway target genes in cells stimulated with cancer cell-derived exosomes. We found that in both PLC/PRF/5 and MHCC-97H cells, the mRNA and protein levels of several target genes, including GLI1, PTCH1, Cmyc, and CyclinD1, were all elevated when Exo was added in the culture medium (**Figures 3E, F**).

**Figure 4:**
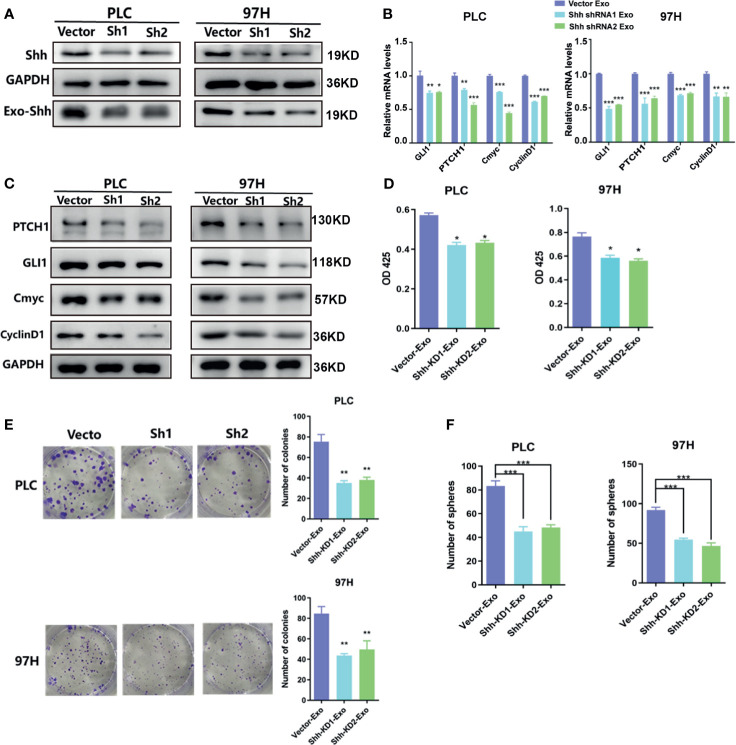
The effect of exosomes isolated from Shh knockdown cells. **(A)** Western blot analysis on the transfection of Shh shRNA lentivirus on the expression of cellular Shh and Exo Shh. **(B)** Quantitative PCR analysis of the mRNA levels, and **(C)** Western blot analysis of the protein levels of hedgehog signaling pathway target genes in PLC/PRF/5 and MHCC-97H cells cultured with exosomes isolated from the culture medium of the same cell line that were transfected with vector or shRNA containing lentivirus. **(D)** Cell proliferations as measured by CCK8 assay, **(E)** colony formation assay, and **(F)** count of sphere formed in PLC/PRF/5 and MHCC-97H cells cultured with exosomes isolated from the culture medium of the same cell line that were transfected with vector or shRNA containing lentivirus. The data are presented as an average of three biological replicates. *p < 0.05, **p < 0.01, ***p < 0.001.

To determine whether the Shh protein carried in these exosomes was responsible for the stimulating effect, we used shRNA to knock down the expression of Shh, and observed its impact. The Western blot analysis demonstrated that two different shRNA-containing lentivirus constructs successfully reduced the expression of Shh in both PLC/PRF/5 and MHCC-97H cells (**Figure 4A**). As a consequence, the Shh levels in the isolated Exo from respective culture medium were also reduced when shRNA-containing lentivirus was transfected into the cells. We isolated the exosomes from the Shh knockdown cells and compared their effect on the Hedgehog pathway and cell growth with the exosomes isolated from the Shh intact cells (vector transfected). As shown in **Figures 4B, C**, when the exosomes isolated from the Shh knockdown cells were added to the culture of the PLC/PRF/5 or MHCC-97H cells, they did not stimulate the expression of Hedgehog pathway target genes as the exosomes isolated from the control cells did in both mRNA and protein levels. Moreover, exosomes from the Shh knockdown cells had reduced ability in stimulating the cell proliferation, colony formation, and sphere formation, compared to the exosome isolated from the control cells (**Figures 4D–F**). These studies indicated that Shh played an important role in PLC/PRF/5 and MHCC-97H cancer development, and the cells secreted the protein into exosome to assert regulation.

### *In Vivo* Experiment

**Figure 5:**
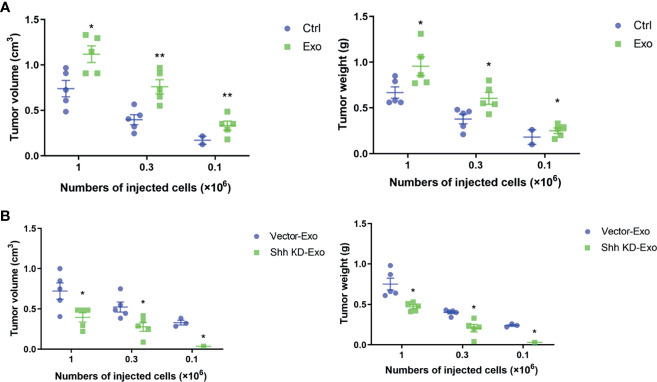
Tumor formations in NOD/SCID mice by PLC/PRF/5. **(A)** The volume and weight of the tumor formed by PLC/PRF/5 subcutaneously injected into NOD/SCID mice with or without mixed with isolated Exo (5 μg/ml) from the same cell culture medium. **(B)** The volume and weight of the tumor formed by PLC/PRF/5 subcutaneously injected into NOD/SCID mice mixed with Exo (5 μg/ml) isolated from culture medium of the vector or Shh shRNA containing lentivirus transfected cells. *p < 0.05, **p < 0.01.

We demonstrated that PLC/PRF/5 and MHCC-97H could secrete Shh containing exosome to regulate the growth of cells. We decided to investigate whether these exosomes influenced tumor formation *in vivo*. PLC/PRF/5 was inoculated subcutaneously into NOD/SCID mice at various numbers mixed with PBS or 5 μg/ml of exosome from its own culture; the tumor formation was observed continuously for 4 weeks. The mice were then euthanized and tumors were removed, measured, and weighted. At the inoculation doses of 1 × 10^6^/mouse and 3 × 10^5^/mouse, tumor was formed in all five mice with cells co-injected with PBS or isolated exosomes. However, the average tumor volumes and weights formed by cells mixed with isolated exosomes were significantly higher than those formed by cells mixed with PBS (**Figure 5A**). At the lowest inoculation of 1 × 10^5^/mouse, however, tumor only formed in 40% (2/5) of the mice when cells were mixed with PBS before injection, while it formed in 100% (5/5) of the mice when cells were mixed with cancer cell-derived exosomes (**Figure 5A**). Similarly, the average weight and volume of the tumors were significantly higher formed by cells mixed with exosome than those formed by control cells.

We then studied whether the exosomes isolated from cells with Shh knockdown would abolish the stimulating effect on tumor formation of the exosomes. We inoculated the NOD/SCID mice with PLC/PRF/5 cells mixed with either exosomes isolated from control or Shh knockdown cells. As shown in **Figure 5B**, at the inoculation doses of 1 × 10^6^ and 3 × 10^5^, PLC/PRF/5 mixed with either exosomes form tumor in all (5/5) mice tested. However, the average volume and weight of the tumors formed by cells mixed with exosomes isolated from untreated cells were significantly higher than those by cells mixed with exosomes isolated from Shh knockdown cells. When mice were inoculated with PLC/PRF/5 at 1 × 10^5^, tumor formed in 60% (3/5) of the mice injected with cells mixed with exosomes derived from normal cells, but none (0/5) with cells mixed with exosomes derived from Shh knockdown cells.

### Clinical Manifestation

**Figure 6:**
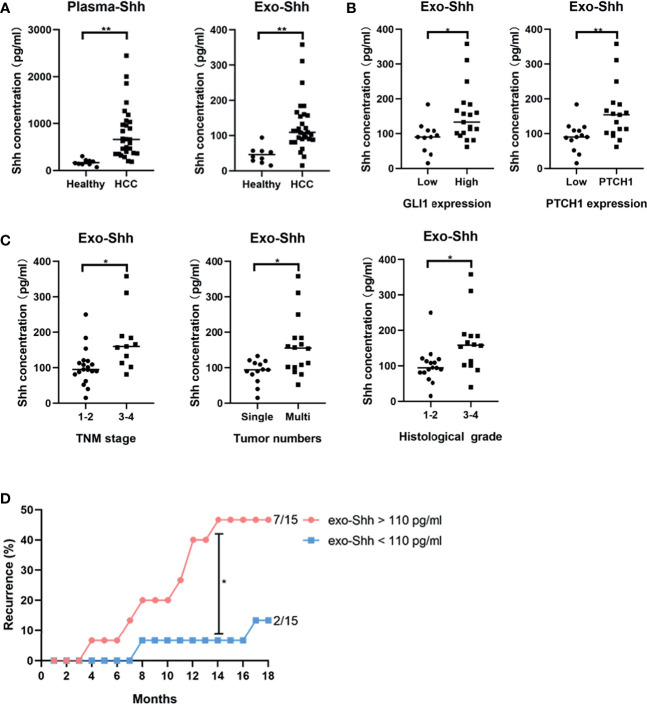
**(A)** Levels of plasma Shh and Exo-Shh isolated from plasma from healthy donors and HCC patients as determined by ELISA. **(B)** Levels of Exo-Shh isolated from plasma of HCC patients grouped based on tissue GLI-1 and PTCH1 levels. **(C)** Levels of Exo-Shh isolated from plasma of HCC patients grouped based on cancer TNM stage, tumor numbers, and histological grade. **(D)** Recurrence of HCC after surgical sections grouped by the Exo-Shh levels. *p < 0.05, **p < 0.01.

Finally, we explored the clinical relevance of circulating Shh levels in HCC patients. We first compared the plasma Shh levels and plasma Exo-Shh levels from 30 HCC patients to 10 normal healthy donors. We found that the average plasma Shh and plasma Exo-Shh levels were both significantly higher in HCC patients than those in healthy donors (**Figure 6A**).

To understand if there is a correlation between circulating Shh levels and the activation of the Hedgehog pathway in HCC, we performed immunostaining on the sections of the tumor tissue for the expression of GLI-1 and PTCH1. Based on the quantitation of the staining, we categorized them into a low-expression and a high-expression group (S3). We then studied correlation between plasma Exo-Shh levels and the expression levels of GLI-1 and PTCH1. As shown in **Figure 6B**, plasma Exo-Shh levels were significantly higher in patients with higher expression levels of GLI-1 and PTCH1 than those with lower levels.

We then analyzed the correlations between the plasma Exo-Shh levels with the clinical characteristics, in terms of TNM stages, number of tumors, and histological grades. As shown in **Figure 6C**, patients with later TMN stages of cancer, multiple tumors, or higher histological tumor grades had significantly higher levels of plasma Exo-Shh than patients with earlier stages of cancer, single tumor, or lower histological grade.

Finally, these 30 HCC patients were followed up for the tumor recurrence after surgery within 18 months. We found that patients with plasma Exo-Shh levels that were higher than 110 pg/ml (*n* = 15) had a recurrence rate of 46.7% within 18 months after surgery, while patients with plasma Exo-Shh levels lower than 110 pg/ml (*n* = 15) had a recurrence rate of 13.3%. Recurrence between the two groups reached a statistical difference at the time point of 14th month post-surgery (46.7% vs 6.7%) (**Figure 6D**).

## Discussion

Exosomes, double-layer membrane-bound EVs, have been the subject of extensive study since its discovery because of its role in the biological process. Many studies have focused on its miRNA contents and their regulatory functions in biological processes and cancer development, including HCC (21, 22). A number of microRNAs (miRNAs), long noncoding RNAs (lncRNAs), and even messenger RNAs (mRNAs) have been shown to be carried by exosomes, asserting regulatory functions to neighboring cells, and can potentially serve as biomarkers for diagnosis and prognosis in HCC (21). Recent studies have also uncovered evidence that proteins can be transported through exosomes and play a role in HCC tumorigenesis. For example, exosomes released from metastatic HCC cell lines, such as MHCC-97L and HKCI-8, have been found to contain pro-tumorigenic RNAs and proteins, which can promote cell migration and invasion upon internalization by hepatocytes (23). Recently, it was reported that lysyl oxidase-like 4 was transferred by exosomes and promoted cell migration *via* the FAK/Src pathway (24). Another protein, alpha-enolase (ENO1), can be transferred by exosomes between HCC cells and upregulates integrin α6β4 through the FAK/Src pathway (25).

In this study, we focused our investigations on the effect of exosome secreted by HCC cells on tumor development through the Shh protein it carried. We demonstrated that HCC cell lines PLC/PRF/5 and MHCC-97H secreted Shh through EVs, preferably in exosome. The Shh containing exosome promoted cell growth, colony formation, and sphere formation. It upregulated the expression of a number of CSC markers. We found that the Hedgehog signaling pathway was activated when the culture medium was supplemented with additional cancer cell-derived exosomes. We demonstrated that when we reduced the Shh levels by shRNA knockdown, the exosomes derived from knockdown cells did not induce Hedgehog signaling pathway or promote cell growth, as the exosomes derived from the untreated cells did. We provided convincing evidence that Shh carried by exosomes was the main signaling molecule in activating the Hedgehog pathway in HCC cells. This is the first illustration of the role of exosome on Hedgehog signaling in HCC tumorigenesis.

Activating the Hedgehog pathway through overexpression of Shh in HCC tissues was confirmed by many studies (10, 26). Cancer cells secreting Hedgehog ligands to activate autocrine signaling of the pathway has also been found in lung cancer (27), prostate cancer (28), and gastrointestinal cancer (29). Some studies also found that cancer cells or stromal cells in cancer microenvironment can secrete Hedgehog protein to promote cancer development in a paracrine fashion (30, 31). Our study provided insights into how Shh can be transported within the tumor to confer its activity, i.e., cancer cells could secrete Shh *via* exosomes that may transport the signaling molecule to a site of greater distance to activate the pathway.

CSC theory has become more attractive in recent years. It is proposed that there exists a small population of tumor cells that carry the characteristics of somatic stem cells and are capable of evolved into different cell types within a tumor. These CSCs are capable of self-renewal, differentiation, metastatic dissemination, and are resistant to traditional therapy. The self-renewal of CSCs is tightly regulated by several signaling pathways, including Wnt/β-catenin, Notch, Hippo, and Hedgehog pathways, although they may be alerted to suit the need for malignancy (32). The origin of these CSCs is not well understood. One theory is that they may originate from a more differentiated cancer cell that acquires stem cell properties through an epithelial-to-mesenchymal transition (EMT) process (33). There is also evidence for a normal stem cell undergoing malignant transformation (34).

Our study showed that HCC cell lines PLC/PRF/5 and MHCC-97H, when cultured with additional Shh containing exosome, had increased sphere formation in cell culture. At the same time, expression levels of several CSC marker proteins also increased. These observations suggested that ligand-dependent activation of the Hedgehog pathway could lead to increased proportion of HCC cells that exhibit the property of CSCs, consistent with the model of cancer cell acquiring stem cell properties. Other studies have also found that activation of the Hedgehog pathway induces the expression of CSC marker CD133 and cytokine IL-6, contributing an important function in the liver acute phase response and in HCC development (35). This may explain the increased ability of PLC/PRF/5 to form tumor in the xenograft model when cells were inoculated with Shh containing exosome. Not only was the inoculation required to form a tumor in mice smaller when PLC/PRF/5 was injected with exosome, the volume and weight of the tumor formed with the same inoculations were also on average larger than those formed with cells injected without exosome.

Transforming growth factor (TGF)-β plays a critical role in the induction of EMT, and it has been found that its expression is elevated in 40% of human HCC tissues (36). A recent study has demonstrated that MHCC-97L and MHCC-97H cell-derived exosomes can induce EMT through the TGF-β/Smad signaling pathway (37). In fact, studies have shown that activated the Hedgehog pathway may induce EMT *via* a number of signaling cascades, including WNT, EGF/FGF, Notch, and TGF-β in a variety of tumors (38). It would be of future interest to investigate cross talk of Hedgehog signaling with other pathways in modulating HCC CSCs.

Lastly, we found that circulating Shh levels in HCC patients were significantly higher than those in healthy people. However, it is the plasma Exo-Shh levels, not total plasma Shh levels (data not shown), that had a positive correlation with the tumor stage and histological grade, as well as the expression levels of Hedgehog pathway components in tumor tissue. Higher plasma Exo-Shh levels were also associated with higher recurrence. These findings suggest that Exo-Shh could serve as a prognostic biomarker.

## Conclusion

**Figure 7:**
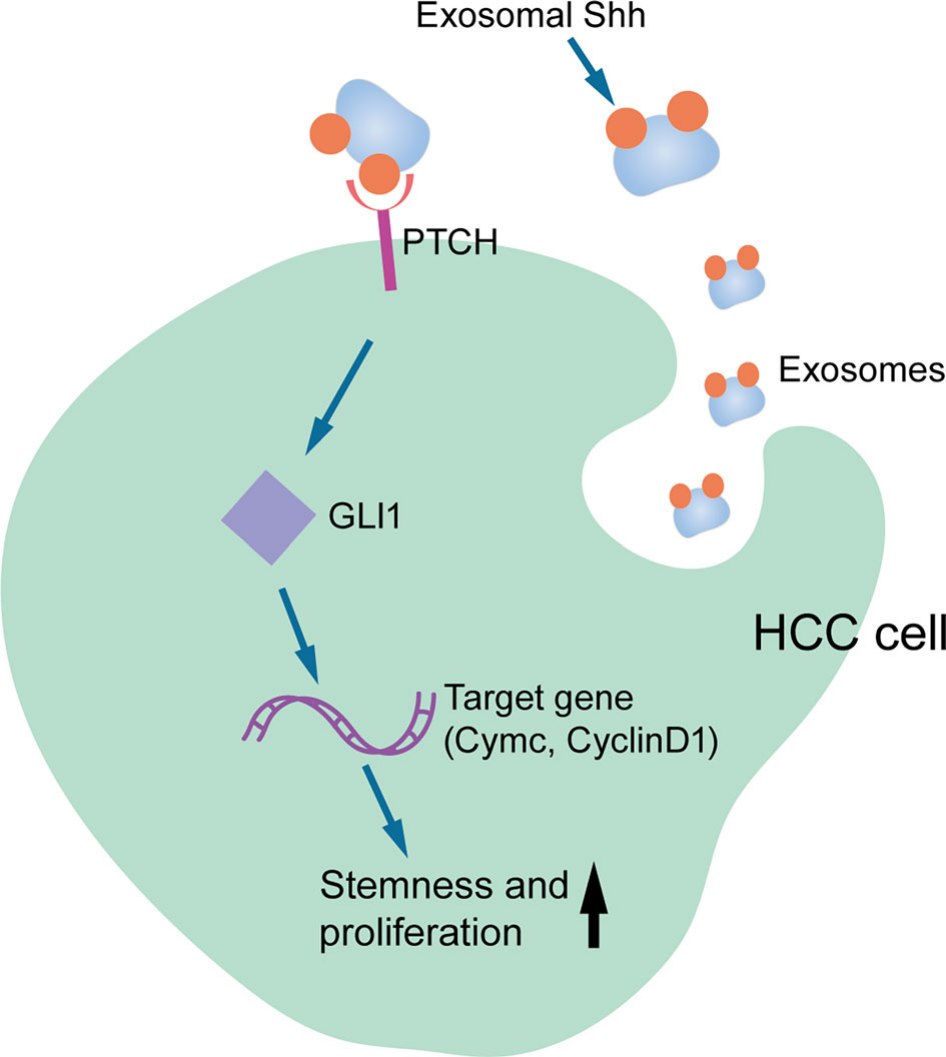
Diagram for the Exo-Shh activating Hedgehog signaling in HCC cells.

In summary, our study provided the first evidence that HCC cells secreted Shh through exosome and this exosome that carried Shh played an important role in HCC tumorigenesis. As shown in **Figure 7**, we proposed that HCC cells secrete Shh protein *via* exosome and Exo-Shh interact with cellular receptor PTCH and induce the activation of Hedgehog signaling in HCC cells to promote tumorigenesis. At present, two Hedgehog pathway-targeted drugs that act on SMO protein, Vimodji and Songyib, have been approved for clinical treatment of basal cell carcinoma. However, in ligand dependently activated tumors, such as gastric cancer, pancreatic cancer, and lung cancer, no clinical benefit has been achieved (39–41). Our findings may point to the possibility of tumor-secreted exosome being a therapeutic target.

Future studies are needed to validate the clinical value of the plasma Exo-Shh as a biomarker for HCC prognosis. The complexity of cancer cell-derived exosomes also requires further analysis to better understand the cross talk of the Hedgehog pathway and other signaling pathways and their roles in tumor progression.

## Data Availability Statement

The original contributions presented in the study are included in the article/**Supplementary Material**. Further inquiries can be directed to the corresponding author.

## Ethics Statement

All human subjects involved in the study have signed informed consent for participation. The experimental protocol was reviewed and approved by the ethics committee of Fudan University. The patients/participants provided their written informed consent to participate in this study. The animal study was reviewed and approved by the ethics committee of Fudan University.

## Author Contributions

LL, JZ, and QZ contributed to the study conception and design, performed main experiments, analyzed data, and drafted this article. YT and CS participated in the other experiments, such as Western blotting. RL, ZM, and JL provided assistance in creating figures and tables. ZW supervised experiments and revised the manuscript. All authors contributed to the article and approved the submitted version.

## Funding

This work was supported by the National Natural Science Foundation of China (81873874 and 82071797), the National Science and Technology Major Project (2017ZX10203205) and Clinical Research Plan of SHDC (No. SHDC2020CR2021B). The funder did not make any substantive contributions to the article.

## Conflict of Interest

The authors declare that the research was conducted in the absence of any commercial or financial relationships that could be construed as a potential conflict of interest.

## Publisher’s Note

All claims expressed in this article are solely those of the authors and do not necessarily represent those of their affiliated organizations, or those of the publisher, the editors and the reviewers. Any product that may be evaluated in this article, or claim that may be made by its manufacturer, is not guaranteed or endorsed by the publisher.
