# Supplementary material for: Cancer Cell-Derived Exosomes Promote HCC Tumorigenesis Through Hedgehog Pathway
Source: Front Oncol. 2021 Oct 7;11:756205. doi: 10.3389/fonc.2021.756205 (PMC8529041; doi:10.3389/fonc.2021.756205)

**Supplementary Figures**

**S1** The total protein levels in MV and Exo isolated from HCC cell lines of PLC/PRF/5 and MHCC-97H were analyzed by Micro BCA Protein Assay Kit (Thermo Scientific) as specified by manufacturer. It was noted that Exo fractions from both cells carried significantly higher amount of protein than MV fractions. Similarly, the nanoparticle tracking analysis revealed that there were more particles presented in Exo fractions than in MV fractions from both cell lines.


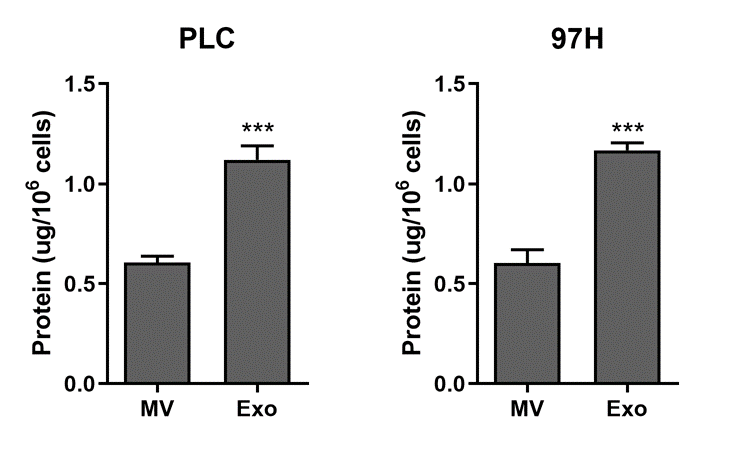

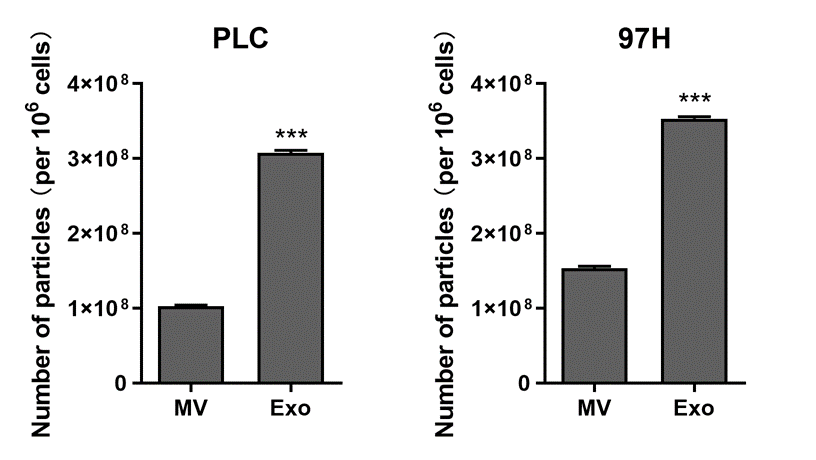


**S2** The levels of hedgehog ligands Shh and Ihh, carried by exosome from HCC cell lines PLC/PRF/5 and MHCC-97H were much higher than those isolated from normal hepatocyte cell line L2 as analyzed by western blot.


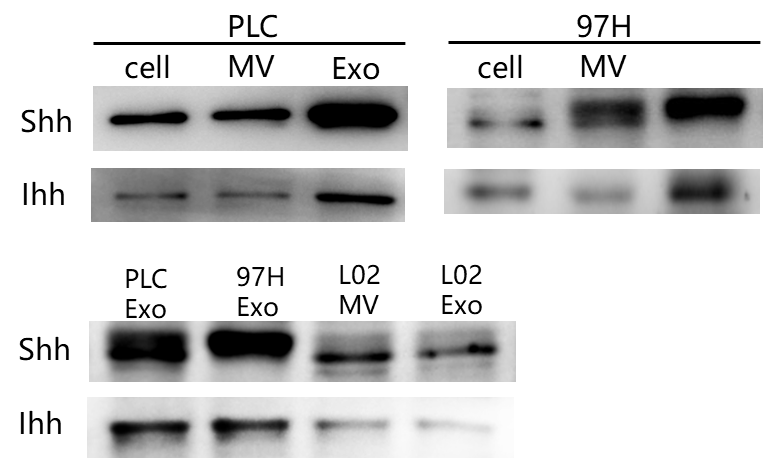


**S3** The immunohistochemistry analysis of expression of GLI1 and PTCH1 in liver tissue section of 30 HCC patients revealed that cancer patients had various expression levels of GLI1 and PTCH1. Expression levels were independently assessed by two pathologists blinded to clinical data. Staining intensity was graded as negative, weak and strong. There was a close agreement on staining intensity between the two pathologists. The patients with negative and week expression were grouped into low expression group, whereas the patients with strong expression were grouped into high expression group. Representative images of stained sections were shown below.


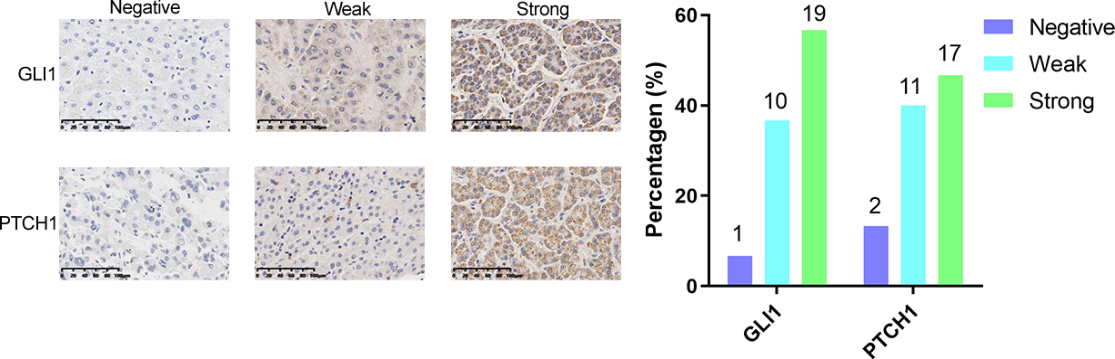

Supplement: Supplementary file 1 [file DataSheet_1.docx]
